# Gel properties of chicken-squilla low-fat mince gel with inulin: Physicochemical properties, microstructure, intermolecular interactions, and formation mechanism

**DOI:** 10.1016/j.fochx.2025.102347

**Published:** 2025-03-17

**Authors:** Lijing Geng, Jing Liang, Dan Wang, Wei Huang, Hang Fu, Mbinga Isequias Elamba Tertulliano, Muhammad Zain Ul Aabideen, Wei Zhou, Quimbamba Silvia Jacinta Calombe, Aqsa Shafique

**Affiliations:** a College of Food and health, Jinzhou Medical University, Jinzhou 121001, China; b Key Laboratory of Molecular Cell Biology and New Drug, Jinzhou Medical University, Jinzhou 121001, China; c Liaoning Province meat processing and quality safety control professional technology innovation center, Jinzhou 121001, China; d College of Clinical Medicine, Jinzhou Medical University, Jinzhou 121001, China

**Keywords:** Inulin, Gel properties, Gel network, Inulin (PubChem SID: 4052344392), Myosin heavy chain (2mys and 6fsa)

## Abstract

This study aimed to examine the relationship of inulin with the chicken-Squilla mince mixture gel (CSMMG). The gel characteristics, microstructure, and intermolecular interactions, and formation mechanism of CSMMG with different inulin were determined. The cooking yield, water-holding property, color value, hardness, elasticity, chewiness, sensory score, microstructural denseness of CSMMG increased as the inulin, maximum at 5 %. Additionally, inulin could promote the formation and hydrophobic interaction of sulfhydryl group, hydrogen bond, disulfide bond, *α*-helix, and reduce the carbonyl content. According to PCA, PC1 had a positive correlation with chewiness, springiness, hardness, hydrophobic interactions, and negative with redness. Inulin obviously enhanced the gelling behavior of myofibrillar protein molecules with a denser and more uniform network structure by the hydrogen bonding between the myosin and inulin. This study will offer a theoretical basis for future research and development of low-fat high-protein meat products.

## Highlights

• Comprehensively tested indicators characteristics of CSMMG with different inulin.
• The effects of the gel-forming capability and the properties by PCA for the first time.
• Solved the sensory quality issue of low-fat and high-protein minced meat.
• The relationship among CY, WHP, MHC, PM et.al and inulin was systematically analyzed for the first time.
• The binding site between inulin and myosin was predicted for the first time.

## Graphical abstract

**Figure:**
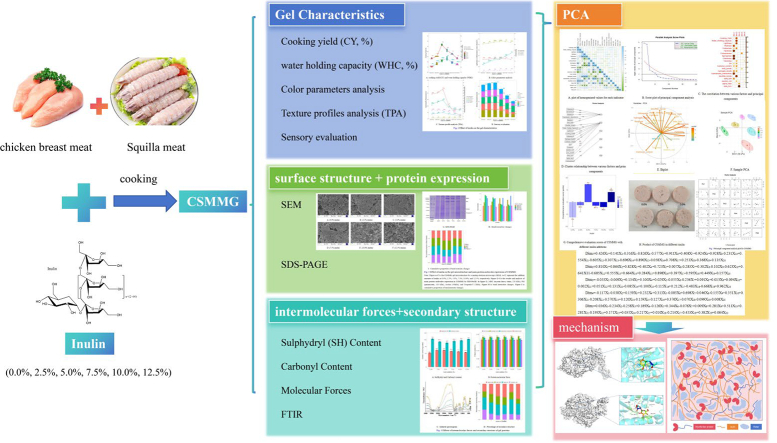

## 1 Introduction

**Fig. 1:**
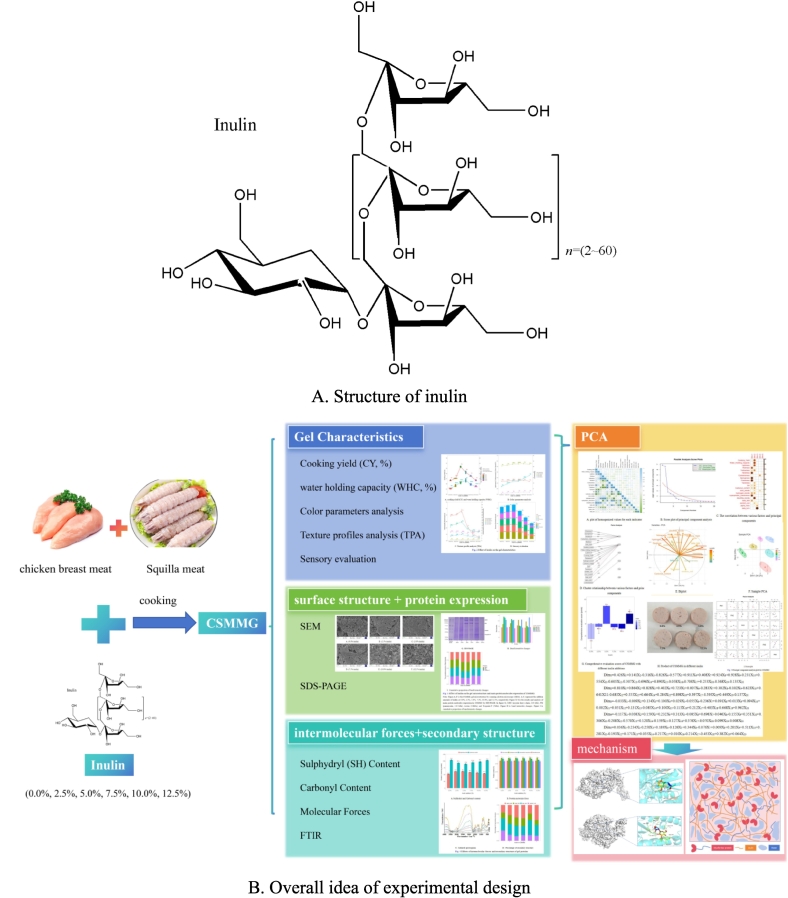
Structure of inulin and overall idea of experimental design.

Inulin found in a wide range of plants is composed of (2 → 1) *β*-d-fructosyl subgroups (Fig. 1A). It therefore belongs to the fructan carbohydrate subgroup and is classified as oligosaccharides or polysaccharides (Ronkart et al., 2007; Mensink^a^ et al., 2015; Mensinkb et al., 2015). Some studies on it have shown that dietary supplementation of inulin could be fermented to generate a series of metabolite by the gut microbiota in the colon, for improving metabolic function and regulating intestinal immunity (Corrêa et al., 2023; Sheng et al., 2023). More recently, it has been proven to be a good water-soluble dietary fiber, and its hydroxyl groups is easy to combine the hydrogen bonds with water strongly to produce molecular associations, thus making the mince to form a more stable emulsion gel structure (Chiavaro et al., 2007; Piao et al., 2023). Moreover, the addition of inulin significantly reduced cooking loss and the content of saturated fatty acids, as well as improved the storage stability of mince (Gu et al., 2024).

Our team have previously developed a new-type low-fat and high-protein nutritional chicken and squilla mixed-meat gel product—chicken squilla mince mixture gel (Abbreviated as CSMMG), which can be further processed into prefabricated food product such as meatballs, sausages, meat cakes, etc. Our previous researches found that this product is very popular from the nutritional perspective and the flavor perspective for the people who pay attention to health preservation in modern times, especially for obese and elderly people. This is because that chicken and squilla mince has low fat and rich protein. Squilla mince has numerous umami-related amino acids and functional peptides to promote the formation of delicious flavours. Nevertheless, the gel-forming capability and the gel properties of CSMMG are very poor due to the low in fat content. So the research on improving the gelation process and gel characteristics of CSMMG and deeply explaining the formation mechanism of gel was very important.

Currently, some studies indicate that ash, moisture, and protein contents of the low-fat chicken sausages were increased with increasing levels of inulin as a fat replacer while water holding capacity (WHC) was similar (Jayarathna et al., 2022). Inulin can inhibit the aflatoxin B_1_ level, and the hardness of sausages is similar to those without added inulin (Chaharaein et al., 2021). In addition, there have been no reports on the application of inulin in shrimp meat at present. Although inulin has a certain effect on meat gel, there isn't a lot of research on the in-depth application of inulin in mixed meat gel, especially in low-fat high-protein mixed meat gel. The team hopes to improve the gel stability of CSMMG by adding some healthy additives like inulin. We preliminarily found that inulin can properly improve the gel strength of CSMMG. We would like to know how inulin can improve the gel effect of mixed minced meat. Therefore, our research team studied the possibility and the mechanism about inulin to properly enhance the gel properties and the gel stability of CSMMG (Fig. 1 B). More importantly, in this study, the effects of inulin on the gel-forming capability and the properties of CSMMG by correlation analysis and principal component analysis for the first time. Especially, it would be interesting to propose a novel interaction mechanism model between inulin and myosin-paramyosin mixture to understand the enhanced gel performance. And finally a comprehensive evaluation model of CSMMG was established for expecting to have the very promising for great and wide food industry applications of inulin in meat.

## 2 Materials and methods

### 2.1 Materials and reagents

Chicken breast meat, salt and potato starch were obtained from Jinzhou RT-Mart supermarket. Fresh squilla (*Oratosquilla oratoria*) were obtained from Jinzhou Guta District Farmers Seafood Complex Market. Inulin (edible grade, 90 % purity, polymerization≈31) was bought from Shaanxi Sweedon Biotechnology Co., LTD. Protein electrophoresis maker (Product number: 26616) was purchased from Lithuanian Fermentas Company. Other reagents were bought from Shenyang chemical reagent factory. All reagents and chemicals used in this study were of analytical grade.

### 2.2 Preparation of CSMMG

Firstly chicken breast meat being removed the fat and connective tissue was cut into about 2 cm × 2 cm × 1 cm blocks. Squilla meat got from fresh squilla (*Oratosquilla oratoria*) was cut into segments about 1 cm in length. Washed the chicken breast meat and Squilla meat, and then mixed them with 2:1 (mass ratio) under 1500 r/min for 3 min. After adding 1.5 % salt, 15 % potato starch and inulin (0.0 %, 2.5 %, 5.0 %, 7.5 %, 10.0 %, 12.5 %) and continuously chopping under 1500 r/min for 3 min, the CSMM was obtained. During the entire process, the center temperature of the mixture maintained below 10 °C by adding 10 % ice water three times separately. CSMM is CSMMG before cooking. Using the two-step method of water bath (heating at 40 °C for 20 min, then 85 °C for 20 min), 60 *g* CSMM which was centrifuged at 4 °C at 400 r/min for 12 min to remove bubbles was heated, and then cooled rapidly for 10 min to form gel. CSMMG was made by heat-induced method. Stored in a 4 °C refrigerator and prepared for subsequent testing and research.

### 2.3 Gel characteristics

#### 2.3.1 The binding ability of water

Cooking yield (CY, %) and water holding capacity (WHC, %) are two common indicators of the water binding ability of meat products during processing. CY was tested and expressed as the percentage weight yield of the heat-induced CSMMG (g) over the original CSMM (g). CY was calculated as the following formula 1. The calculation formula of WHC is as the following formula 2.

(1) $$\mathrm{CY}\left(\%\right)=\frac{m_{2}}{m_{1}}\times 100$$

Where: *m*_1_ is the weight (g) of CSMM after cooking, *m*_2_ is the weight (g) of CSMMG before cooking.

(2) $$\mathrm{WHC}\left(\%\right)=\frac{m_{4}}{m_{3}}\times 100$$

Where: *m*_3_ represents the precentrifugation weight (g) of the circular slice shape CSMMG samples (the thickness about 0.1 cm and the bottom diameter about 3 cm), *m*_4_ indicates the weight (g) of CSMMG after centrifugation (6000 r/min for 20 min at 4 °C, CSMMG samples wrapped with three layers of filter paper).

#### 2.3.2 Color parameters analysis

The color characteristics [L* (lightness), a* (redness), and b* (yellowness) values] of CSMMG samples were measured three times for each sample by CR-400 colorimeter (Konica Minolta Company, Japan). The whiteness value (*W*, Formula 3) was calculated as the following formula 3.

(3) $$W=100-\sqrt{{\left(100-L^{*}\right)}^{2}+{a^{*}}^{2}+{b^{*}}^{2}}$$

#### 2.3.3 Texture profiles analysis (TPA)

Texture profiles (hardness, springiness, cohesiveness, gumminess, and chewiness) of CSMMG samples were determined by TA-XT plus texture analyzer (Stable Micro System Inc., UK). For this purpose, a universal cylindrical probe (70 mm diameter) was employed. The CSMMG sample was cut into a cylinder (3 cm diameter×2 cm thick) and subjected to two cycles (3 s holding time) of 50 % compression at a pre-test speed of 2.00 mm/s, test speed of 1.00 mm/s, post-test speed of 2.00 mm/s with 5.0 g trigger force.

#### 2.3.4 Sensory evaluation

According to the procedures which we have revised, the sensory evaluation system of each CSMMG was performed though a ten-member experienced sensory evaluators (Zhang et al., 2021). Each CSMMG was evaluated by certain sensory descriptive analysis (CSDA, ESM-Table. 1) according to the following 10-point score (1–3: low perceptible; 4–7: medium intensity; 8–10: extremely intense; a maximum score of 50 points). The evaluated attributes were interior color (color intensity, glossiness and color uniformity), uniformity degree of the cutting surface (flatness, stomata number and juiciness), flavours (cooked chicken flavor, cooked shrimp flavor and other flavours), taste (elasticity and chewiness) and overall acceptability. The sensory evaluation was approved by the Ethics Com mittee of Jinzhou Medical University and all panelists provided informed written consent before the experiments.

### 2.4 Detection of surface structure and main protein molecules expression in CSMMG

#### 2.4.1 Scanning electron microscopy (SEM)

CSMMG samples (2 mm × 2 mm × 2 mm) were mixed with 2.5 % glutaraldehyde at 4 °C for 12 h. Rinsed three times with 0.2 mol/L pH 7.2 phosphate buffer for 15 min each time. Subsequently the samples were dehydrated using gradually increasing concentrations ethanol (30 %, 50 %, 70 %, 90 %, 95 % and 100 %) for 15 min each time. CSMMG dried for 24 h in FD-1B-80 vacuum freeze dryer (Beijing Bokang Experimental Instrument Co., LTD, China) was adhered to the double-sided adhesive of conductive carbon film and were sputtered gold on the sample stage of ion sputtering instrument. Finally, the microstructure of CSMMG was observed by KYKY-EM 8000F SEM (Beijing Zhongke Keyi Co., Ltd., China) and photographed.

#### 2.4.2 SDS-page

Proteins samples extracted from 3 g CSMMG was separated by sodium dodecyl sulfate-polyacrylamide gel electrophoresis (SDS-PAGE). Stained with Coomassie brilliant blue solution for 180 min, and decolorized with methanol-glacial acetic acid solution (*V*_methanol_: *V*_glacial acetic acid_: *V*_pure water_ = 1:2:17) until the band was clear, and photographed. The band intensities were determined by the *adobe photoshop CC* software (Adobe Systems Incorporated, USA).

### 2.5 Detection of intermolecular forces and secondary structure of myofibril protein in gel

#### 2.5.1 Preparation of myofibril protein solution (MPs)

Myofibrillar protein was isolated from CSMMG according to the method of Barido and Lee (2021). Briefly, CSMMG was homogenized with 10 volumes of isolating buffer [20 mmol/L phosphate buffer (pH = 7.0), 1 mmol/L ethylene glycol-bis (2-aminoethyl ether)-N,N,N′,N′-tetraacetic acid (EGTA), 0.1 mol/L NaCl)]. The homogenate mixture was centrifuged at 5000 r/min for 15 min (4 °C). The supernatant was discarded, and the residual pellets were resuspended in the isolating buffer. The above steps was repeated three times. The collected supernatants were solubilized by 25 mmol/L buffer (0.6 mol/L NaCl, pH 7.0) and were filtered with 4 layers of gauze, and the filtrate was the isolated CSMMG myofibril protein solution (MPs). Stored in a 4 °C and used within 48 h.

#### 2.5.2 Effects of intermolecular forces of MPs.

##### 2.5.2.1 Determination of Sulphydryl (SH) Content.

The methodwas used to measure SH content (Yang et al., 2021). Briefly, 10 mL of 50 mmol/L Tris-HCl buffer (1 mmol/L EDTA, 6 mol/L guanidine hydrochloride, pH 8.3) and 0.1 mL 10 mmol/L 5,5′-dithiobis (2-nitrobenzoic acid) (DTNB) were added to 1 mL of MPs, followed by the incubation at 25 °C for 30 min. The content of SH (formula 4) was determined at 412 nm using a U-T6 UV–visible spectrophotometer (Yi Pu Instrument Manufacturing Co., China) with a molar extinction coefficient of 13,600 L/(mol·cm).

(4) $$\mathrm{SH}\;\text{content}\left(\text{nmol}/\mathrm{mg}\;\text{protein}\right)=\frac{A_{412}\times 10^{6}}{13600\times C}$$

Note: *A*_412_ was the absorbance at 412 nm. 10^6^ was molar base units. 13,600 is the molar extinction coefficient, L/(mol·cm). *C* was the measured protein concentration, mg/mL.

##### 2.5.2.2 Determination of carbonyl content

The carbonyl content was determined as described by Chen et al. (2024) with slight modifications. In brief, 1 mL MPs was mixed with 50 mL 20 mmol/L 2,4-dinitrophenylhydrazine (DNPH) for 1 h in the dark at 25 °C. Shook once every 25 min. The mixtures were added with 50 mL 20 % trichloroacetic acid (TCA) and centrifuged at 4 °C 9000 r/min for 20 min. Then, the precipitate was mixed three times by 10 mL ethyl acetate/ethanol. The sediment was obtained and redissolved using 10 mL 6 mol/L guanidine hydrochloride under 37 °C water bath for 25 min, followed by centrifugation at 4 °C 9000 r/min for 8 min. The absorbance at 370 nm was measured to calculate the carbonyl content (formula 5).

(5) $$\mathrm{carbonyl content}\left(\text{nmol}/\mathrm{mg}\;\text{protein}\right)=\frac{A_{370}\times 10^{6}}{22000\times C}$$

Note: *A*_370_ was the absorbance at 370 nm. 10^6^ was molar base units. 22,000 is the molar extinction coefficient, L/(mol·cm). *C* was the measured protein concentration, mg/mL.

#### 2.5.3 Effects of secondary structure of gel proteins

##### 2.5.3.1 Molecular forces

The Molecular Forces was determined as described by Chen et al. (2023) with slight modifications. 4.0 g CSMMG were homogenized (3000 r/min, 2 min) in the following chemicals, including S1 (0.6 mol/L NaCl), S2 (0.6 mol/L NaCl +1.5 mol/L urea), S3 (0.6 mol/L NaCl +8 mol/L urea), S4 (0.6 mol/L NaCl +8 mol/L urea +0.5 mol/L β-Mercaptoethanol). Then the obtained homogenates were stored (4 °C, 1 h) and centrifuged (4 °C, 9000 r/min, 20 min). Protein concentration in supernatants was measured by The concentrations of the dissolved protein were measured adopting the Coomassie blue protein method, and differences in protein concentrations were determined by the presence of molecular forces (ionic bonds, hydrogen bonds, hydrophobic interactions, and disulfide bonds) in CSMMG. Results are expressed as mg/mL (soluble protein/ homogenate).

##### 2.5.3.2 FTIR

Each FTIR (Fourier transform infrared spectroscopy) samples were prepared the same as aforementioned in CSMMG. The secondary structures were determined using a Fourier transform infrared spectroscope (Y-Great20 Fourier infrared spectrometer, Zhongke Ruijie Technology Co., Ltd., China). Spectral data were accumulated using 32 acquisitions with 4 cm^−1^ resolution at 4000–400 cm^−1^ scanning wa*v*elength (Zhao et al., 2018). The secondary structure content of proteins was calculated by peak fitting with Peak Fit v 4.12 software.

### 2.6 Principal component analysis (PCA)

PCA is a linear dimensionality reduction statistical method for useful visualizing and interpreting large data sets by forming fewer composite variables (Liu et al., 2024; Zhao et al., 2018). The factor analysis was conducted to derive the interaction between gel characteristics (cooking yield, water holding capacity, lightness, redness, yellowness, hardness, cohesiveness, springiness, chewiness, tackiness) and main protein molecules (sulfhydryl, carbonyl content, ionic bonds, hydrogen bonds, hydrophobic interactions, disulfide bonds, beta fold, random coil, alpha helix, beta turn) in CSMMG with different inulins. No transformation rotated the factors by *SPSS 26.0* software (SPSS Inc., Chicago, IL, USA) and *R* (the R Foundation on Mastodon, BlueSky, or LinkedIn.).

### 2.7 Molecular docking

Inulin was bound with myosin by *AutoDock 1.5.7* software package (Molecular Graphics Laboratory). The myosin 3D model was obtained from PDB (*https://www.rcsb.org/*). 2mys and 6fsa was selected as the model of myosin heavy chain in chicken and shrimp, separately. The inulin 3D structure (PubChem SID: 405234439) was obtained from the National Library of Medicine (*https://www.ncbi.nlm.nih.gov/*). Before docking, myosin and inulin were pretreated with *AutoDock Tools* to obtain pdbqt format files. The receptor protein and ligand small-molecule structures were removed from water molecules and added any absent hydrogen atoms to the myosin by *AutoDock Tools*. The *AutoDock Tools* was used to dock myosin with inulin. After calculation, the system automatically generated the docking results according to the binding energy. Select the optimal docking result and save it as a PDB format file for subsequent analysis. Finally, the 3D resulting docking patterns of myosin and inulin were analyzed visually using *PyMoL 2.2.0* software (Schrodinger, Inc., USA).

### 2.8 Statistical analysis

Data expressed as mean ± standard ($\bar{x}\pm S$) and were analyzed from at least three independent experiments (*n* = 3) by *SPSS 26.0* software (SPSS Inc., Chicago, IL, USA). *Homogeneity test* of variance was performed and then followed by *one-way ANOVA* (analysis of variance) for comparison of means of multiple samples. The further tests about multiple comparisons between groups were performed by *Games-Howell method* for data (the results of chewiness, thiol groups, hydrophobic interactions and disulfide bonds) with heterogeneity of variance and *Dunn's Test* for data (other results) with homogeneity of variance after ANOVA. *P ≤* 0.05 was considered statistically significant. Different lowercase letters represent significance differences at *P* ≤ 0.05 and different uppercase letters represent extremely significant differences at *P* ≤ 0.01.

## 3 Results and discussions

### 3.1 Effect of inulin on the gel characteristics of CSMMG

#### 3.1.1 The binding ability of water

**Fig. 2:**
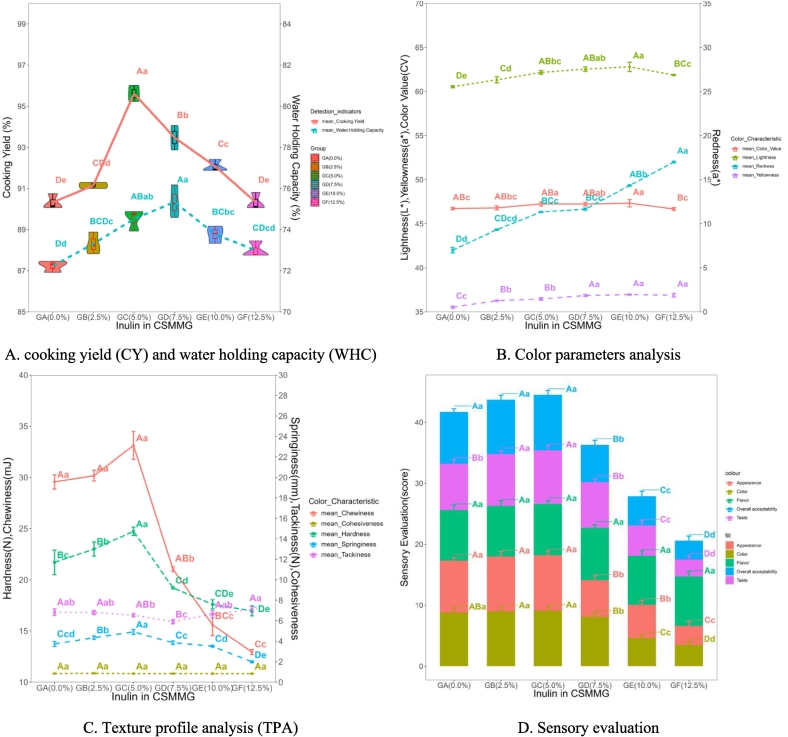
Effect of inulin on the gel characteristics.

Cooking yield (CY) is an important index to judge the formation of protein network structure in the process of meat gel formation. The measure of water holding capacity (WHC) of meat products is a very important attribute examining meat quality variation. Changes of CY and WHC in CSMMG made in different inulin addition are displayed in Fig. 2 A. On the whole, CY and WHC of CSMMG with the increase of inulin addition showed a trend of first increasing and then decreasing. In Fig. 2 A, CY of CSMMG significantly increased under less than 5.0 % inulin (*P* < 0.01), because low-addition inulin rich in polyhydroxyl groups could probably be cross-linked with proteins to form a dense and stable three-dimensional network structure (Sawant et al., 2024). WHC of CSMMG gradually increased when the inulin addition amount was 0.0 % ∼ 7.5 %. Because improvement of gel network structure could increase the binding capacity and the retention capacity of gel to water. It would make numerous water molecules to be subsequently retained even under external forces. As the inulin addition amount exceeded 5.0 %, CY of CSMMG significantly decreased (*P* < 0.05). Maybe too much inulin (a water-soluble dietary fiber) would compete for moisture with the protein in the gel system (CSMMG). This competition hindered the cross-linking between myofibrillar proteins, making it easier to seep out water molecules. It resulted the decrease of CY (*P* < 0.01). At this time, the proportion of free water inside the gel increased. When CSMMG was subjected to external force, the water loss was serious and the value of WHC decreased. The results showed that the quality of CSMMG was improved after adding inulin. The results of cooking rate (CY) and water holding capacity (WHC) suggested that the ability of meat products to bind water and the mixture meat gel product quality were strong and good. Some of our result about WHC is similar to the research findings of Jayarathna, et al. (Jayarathna et al., 2022).

#### 3.1.2 Color parameters analysis

Color is one of the very important indicators for evaluating the sensory quality of meat gel. The influence of 0 % ∼ 10.0 % inulin on the color parameters (*W*-value, L*-value, a*-value and b*-value) of CSMMG are shown in Fig. 2 B. Inulin increased the textural lightness (L*-value) and yellowness (b*-value) of meat products (*P* < 0.01). It means CSMMG to be more shiny and lubricated (Alaei et al., 2018). During the CSMMG heat treatment process, the fructose residue and other substances were produced by inulin hydrolysis. They reacted with the meat protein to enhance the degree of Maillard reaction and produced black-like substances, resulting in the increase of b*-value. The a*-value (redness) increased very significantly (*P* < 0.01). It indicates that the antioxidant properties of inulin had a certain protective effect on myoglobin and hemoglobin in meat. The *W*-value (whiteness) of CSMMG gradually increased and stabilized at last (*P* < 0.05). It might be due to inulin became white creamy gel during processing. *W*-values and L*-values of CSMMG decreased significantly when the inulin addition was higher than 10.0 % (*P* < 0.05). It approximately also might be caused by the excessive Maillard reaction. In this paper, low level of inulin increased the L*-value, b*-value, a*-value and W-value of meat products, but other results show that inulin can enhance the L*-value and a*-value and reduce the b*-value of pork and chicken meatballs (Montoya et al., 2022). Inulin in blended surimi produced from silver carp and bay scallops can enhance the L*-value, a*-value and reduce the b*-value, W-value (Geng et al., 2024). This indicates that the influence of inulin on the color of meat products from different materials and different processing techniques is different.

#### 3.1.3 Texture profile analysis (TPA)

The measured and calculated parameters by texture profile analysis (TPA) are supposedly objective measures for testing the texture properties. For example, chewiness, cohesiveness, hardness, springiness and tackiness. The results are shown in Fig. 2 C. Among all of the determined TPA parameters, hardness and springiness displayed significantly improved (*P* < 0.01) in Group GC (inulin 5.0 %) compared with the no-inulin control group GA (0.0 %). Hardness represents the force required to compress CSMMG. Chewiness indicates the capacity of CSMMG being chewed to the state adequate for swallowing. Springiness reflects the capacity of CSMMG to recover its original shape and size after the removal of the external forces. It indicated that the long chain structure of inulin could crosslink with myofibrillar protein to form a dense three-dimensional network gel structure during the heatment (Piao et al., 2023).

However, GSMMG had the significant decrease (*P* < 0.01) in hardness, springiness and chewiness, while no change (*P* > 0.05) in tackiness under the 12.5 % inulin. It reveals that excessive inulin could destroy the crosslinking with myofibrillar protein and the stability of the gel. In this case, excessive inulin was easier to combine with water to form a softer gel. The significant changes in these indicators indicated that the appropriate inulin could be beneficial to combine with protein to promote the formation of gel, while excessive addition blocked the cross linking between proteins. Lastly appropriate inulin combined with water to form a soft gel properties of CSMMG. Therefore, the stickiness of the composite meat gel reached the lowest value when the amount of inulin was 7.5 % (*P* < 0.05), and then gradually increased with inulin concentration. Among all TPAs, the cohesiveness of CSMMG showed no significant effect with the addition of inulin (*P* > 0.05). Our results showed that the appropriate amount of inulin made gel more compact in gel structure, brighter in gel color, more stable in appearance structure, more elastic in taste and higher in acceptability, this high acceptance of inulin similar to the pork and chicken meatballs adding inulin (Montoya et al., 2022).

#### 3.1.4 Sensory evaluation

From the data results in Fig. 2 D, it was clearly inferred that the amount of inulin added could affect the total sensory score of CSMMG (*P* < 0.01). For the total sensory score, CSMMG with 5.0 % inulin got the maximum average score and there was no significant difference (*P* > 0.05) compared to Group 2.5 % inulin. After comparing these two groups with the other groups separately, the differences were extremely significant (*P* < 0.01). The groups with higher concentration (7.5 %, 10.0 %, 12.5 %) of inulin significantly reduced the color, appearance, taste and overall acceptability scores of CSMMG (*P* < 0.05). However, inulin had no significant effect on the flavor sensory score of gel (*P* > 0.05). This possibly was due to the fact that inulin is a natural plant hydrophilic colloid with no special odor, which could not introduce peculiar or strange odors and taste to CSMMG. Results showed that the appropriate amount of inulin made gel more compact in gel structure, brighter in gel color, more stable in appearance structure, more elastic in taste and higher in acceptability. But too excessive inulin was not conducive to the formation of gel and reduced the sensory quality of gel eventually. The sensory evaluation was approved by the Ethics Com mittee of Jinzhou Medical University and all panelists provided informed written consent before the experiments.

### 3.2 Interaction between inulin and main protein molecules in CSMMG

#### 3.2.1 Scanning electron microscopy (SEM)

**Fig. 3:**
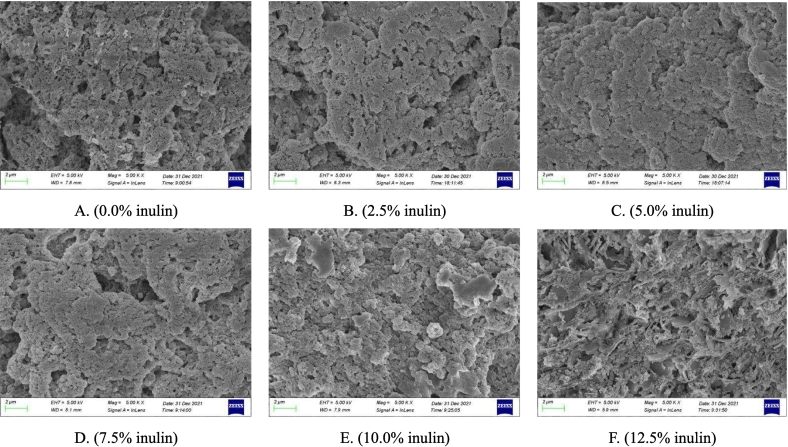

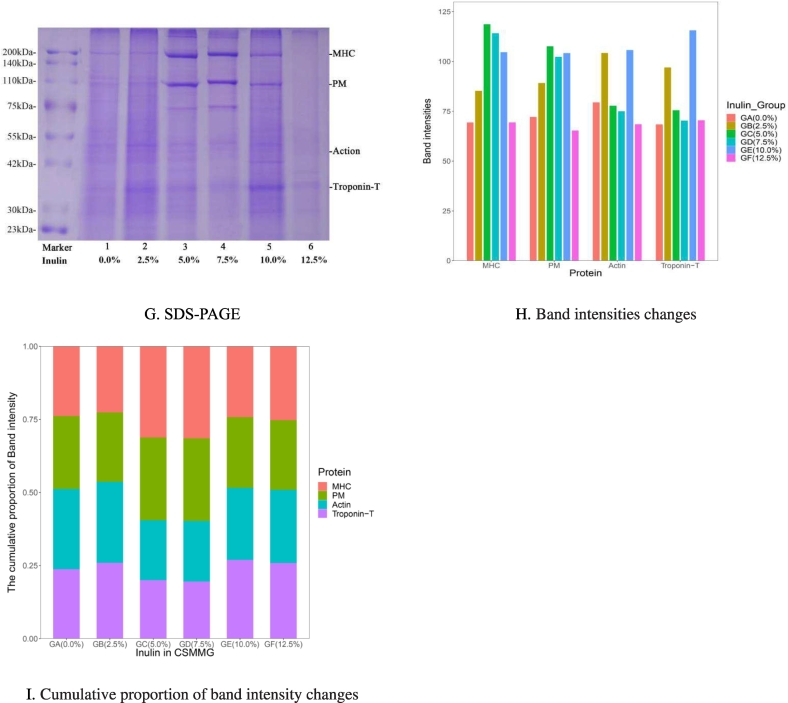
Effect of inulin on the gel microstructure and main protein molecules expression of CSMMG.

Note: Fig. A ∼ F is the CSMMG gel microstructure by scanning electron microscopy (SEM). A ∼ F, represent the addition amounts of inulin at 0.0 %, 2.5 %, 5.0 %, 7.5 %, 10.0 %, and 12.5 %, respectively. Fig. G ∼ I is the results and analysis of main protein molecules expression in CSMMG by SDS-PAGE. In fig. G, MHC (myosin heavy chain, 220 kDa), PM (paramyosin, 110 kDa), Action (45 kDa), and Troponin-T (7 kDa). Fig. H is band intensities changes. Fig. I is cumulative proportion of band intensity changes.

Currently, many studies have shown the relationship between gel properties and gel microstructures is close. It is related to protein aggregates in meat products (Fang et al., 2023; Yan et al., 2021). In this study, scanning electron microscopy (SEM) for 3D microstructure images was used to investigate the microstructures of CSMMG (Fig. 3 A ∼ F). The results can reveal the relationships between the microstructures of CSMMG and different inulin adding amounts. The Fig. 3 A showed that the microstructures of CSMMG without inulin (0 %) was loose and rough, and the existence of protein aggregates could be obviously observed on the surface. And the microstructure gradually became uniform and the pores significantly decreased with the addition of inulin (Fig. 3 B ∼ C). However, the microstructure of CSMMG with excessive inulin was gradually loosened with large pores and the formation of large chamber structures promoted (Fig. 3 D ∼ F). These results revealed that the appropriate amount of inulin could combine with proteins and promote cross-linking between proteins, especially myofibrillar molecules. The combination could transform the filamentous cross-linking structure into a more dense cluster cross-linking structure. Lastly the cross-linking formed a dense and stable three-dimensional network structure. It made the CSMMG structure more compact. This is consistent with the cooking yield and WHC results we previously tested. More inulin would cause it being aggregated and attached to the surface of gel. Inulin could reduce the degree of mutual entanglement and cross-linking of gel network by competing for water with protein. Thus, the gel quality of CSMMG, like the elasticity, chewiness and other sensory indicators were reduced ultimately consistent with our research data. In general, inulin as a water-soluble dietary fiber effectively reduced the gel porosity and enhanced the water retention ability by distributing in the CSMMG system uniformly (Piao et al., 2023).

#### 3.2.2 SDS-page

The results of main protein molecules expression in CSMMG by SDS-PAGE showed the major myofibrillar proteins in CSMMG were myosin heavy chain (MHC) with molecular weight of 220 kDa, paramyosin (PM, 110 kDa), actin (45 kDa), troponin-T (37 kDa) and other proteins (Fig. 3 G). The data analysis of protein expression indicated that the MHC and PM protein products in lanes 3,4 and 5 were increased than lanes 1,2 and 6, and the content change of Actin and Troponin-T were Almost unchanged (Fig. 3 H and Fig. 3 I). These results indicated that inulin especially at 5.0 % ∼ 7.5 % could promote the cross-linking and aggregation of MHC and PM proteins and changed the protein gel properties in CSMMG. Excessive inulin (lane 6 of Fig. 3 H and Fig. 3 I) might hinder protein cross-linking and promoted protein intramolecular bonding to cause protein degradation during heating. All of these would lead to a decline in gel properties. Appropriate inulin could fully integrate with the proteins of chicken and shrimp meat to form a dense and stable three-dimensional network structure for the better retain moisture in meat products, as shown in the electron microscopy and SDS-PAGE results in the paper. Studies have shown that inulin can increase the total protein content (Jayarathna et al., 2022), but there are currently no reports on the effect of inulin on SDS-PAGE in any meat.

### 3.3 Effects of intermolecular forces and secondary structure of gel proteins

#### 3.3.1 Sulfhydryl (SH) content

**Fig. 4:**
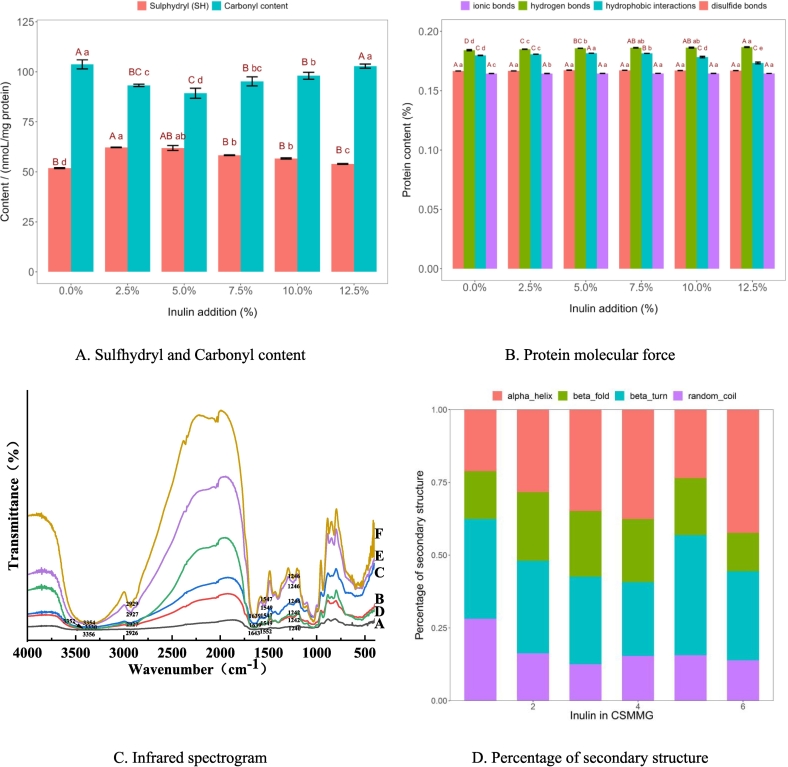
Effects of intermolecular forces and secondary structure of gel proteins.

Fig. 4 A is the variation of SH and carbonyl content which represents the changes of intermolecular forces. It can be seen that the SH content in the gel increased and reached the maximum value at 2.5 % and 5 % inulin (*P* < 0.01). It indicated that inulin could promote the more SH in proteins to be exposed. The increase of the SH content contributed to the formation of the S—S bonds in CSMMG. Finally, the stable and dense protein gel structure was formed (Zhao et al., 2023).

#### 3.3.2 Carbonyl content

The carbonyl content of the proteins gradually decreased significantly with 0.0 % ∼ 5.0 % inulin (Fig. 4 A) (*P* < 0.05). Then increased with 5.0 % ∼ 12.5 %. Probably because more inulin made the distant of protein molecules futher, which affected the protein cross-linking. Next, proteins were more susceptible to damage during the heating process.

#### 3.3.3 Molecular forces analysis

The formation of protein gel is related to hydrogen bonds, hydrophobic interactions, S—S bonds and ionic bonds. When these molecular forces are fully combined, a three-dimensional network structure is formed in the gel, and its density and order can determine the properties of the protein gel (Wu et al., 2022). The effect of inulin addition on the molecular forces in CSMMG is shown in Fig. 4 B. The results showed that the addition of inulin had no significant effect on the ionic bonds, probably because inulin was one of neutral polysaccharides and could not carry any charge (Li et al., 2023). The content of hydrogen bonds increased significantly following with inulin (Fig. 4 B). It indicated that inulin enhances the gel properties by promoting the formation of hydrogen bonds and reducing the interaction between proteins and water (Wang et al., 2020). So the strengthening of hydrogen bonds is beneficial to the formation of gel structures. However, a lot of inulin could increase viscosity of gel and hinder protein cross-linking, which negatively affected the gel quality. The content results of hydrophobic interactions and S—S bonds showed the same tendency of increasing firstly and then decreasing with different inulin contents. At the low inulin concentration, hydrophobic interactions and S—S bonds could promote the formation of the strong three-dimensional network gel structure between protein molecules (Zhang et al., 2023). In contrast, because of the polyhydroxyl and hydrophilic of inulin, it in high concentration reduced the hydrophobic interactions and S—S bonds content which was not conducive to the formation of gel.

#### 3.3.4 FTIR

The FTIR spectrum was examined to gain a better understanding of the relationship between the chemical bond of CSMMG and inulin (Fig. 4C). The dominant and wide-ranging spectrum characteristic detected in the wavenumber region of 500–4000 cm^−1^ is explained by the vibrational modes resulting from the vibrational-vibrational interaction. The infrared spectra of proteins and peptides are mainly concentrated in the amide A band (3500–3000 cm^−1^), amide I band (1700–1600 cm^−1^), amide II band (1550–1530 cm^−1^), amide III band (1330–1220 cm^−1^), and amide B band (around 2925 cm^−1^).

The amide A band exhibits N—H and O—H stretching vibration absorption peaks, which are important regions for analyzing the interaction between proteins and water molecules. In the Fig. 4 C, the band absorption peak of amide A displayed an increasing firstly and then decreasing trend with the inulin, like shifting from 3356 cm^−1^ to 3330 cm^−1^ and eventually back to 3354 cm^−1^. It suggested that the interaction between protein and water increased initially, most likely because inulin enhanced hydrogen bonding and promoted protein and water combination at low content levels (Ji et al., 2021). Amide *I* (1700–1600 cm^−1^), which was caused by C=O stretching vibrations is commonly used to analyze the stability of protein secondary structure and is an important area for analyzing the interaction between polysaccharides and proteins. Protein secondary structures, including *α*-helix, *β*-fold, *β*-turn and random coil structure, can be demonstrated in the amide I band (Wu et al., 2011). In the Fig. 4 C, the band absorption peaks of amide I moved from 1643 to 1639 cm^−1^. It indicated that the increase of inulin led to the conversion of random coil structure to β-folding in CSMMG. Amide II (1550–1530 cm^−1^) band is mainly generated by N—H bending vibration. The main absorption peaks of amide II spectrum moved in the blue shift from 1552 to 1547 cm^−1^ in the Fig. 4 C. It displayed the whole dynamic changes of hydrogen bonds during the protein folding stage in CSMMG.Furthermore, the amide III band is linked to the *α*-helix, *β*-turn, random coil, and *β*-fold structures (Wu et al., 2023). The characteristic absorption peak of amide III band was found to be displaced from around 1240 cm^−1^ to 1246 cm^−1^. It showed that the *β*-folding pattern had dynamically changed in the protein secondary structure in CSMMG. This result confirmed the results of amide I. The findings showed that a denser and more stable structure of complex minced meat gels had been made by the electrostatic interactions between the amino groups of the protein and the carboxyl groups of inulin.

#### 3.3.5 Percentage content analysis of protein secondary structure

The protein secondary structure of CSMMG which were affected by different inulin additions is shown in Fig. 4 D. The percentage of *α*-helix and *β*-folding increased and the one of two unstable protein structures *β*-turn and random coil structure decreased, when 0.0 % ∼ 5.0 % inulin was added. Inulin facilitates the conversion of α-helix to other secondary conformations during the surimi gel fortification (Piao et al., 2023), different from in CSMMG. Although the secondary structure changes of proteins are different, the abundant -OH groups at the surface of inulin can effectively boost and enhance the chemical forces within proteins to reinforce and strengthen the gel network in CSMMG. This suggested that the intermolecular hydrogen bonding connections between peptide chain molecules were improved, which encouraged the cross-linking and aggregation between protein molecules and improved the gel quality. Additionally, inulin could partially inhibit the oxidative reaction of proteins during processing. These was helpful in stabilizing the protein structure and enhancing the quality of the gel.

### 3.4 Correlation analysis and principal component analysis of gel quality indexes

#### 3.4.1 Analysis between gel properties and related indicators

**Fig. 5:**
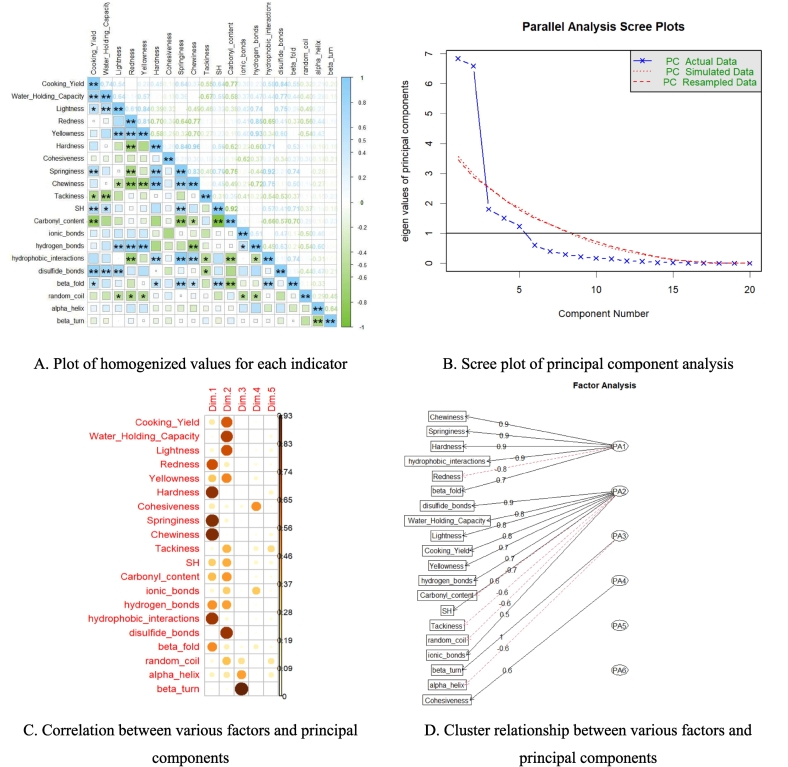

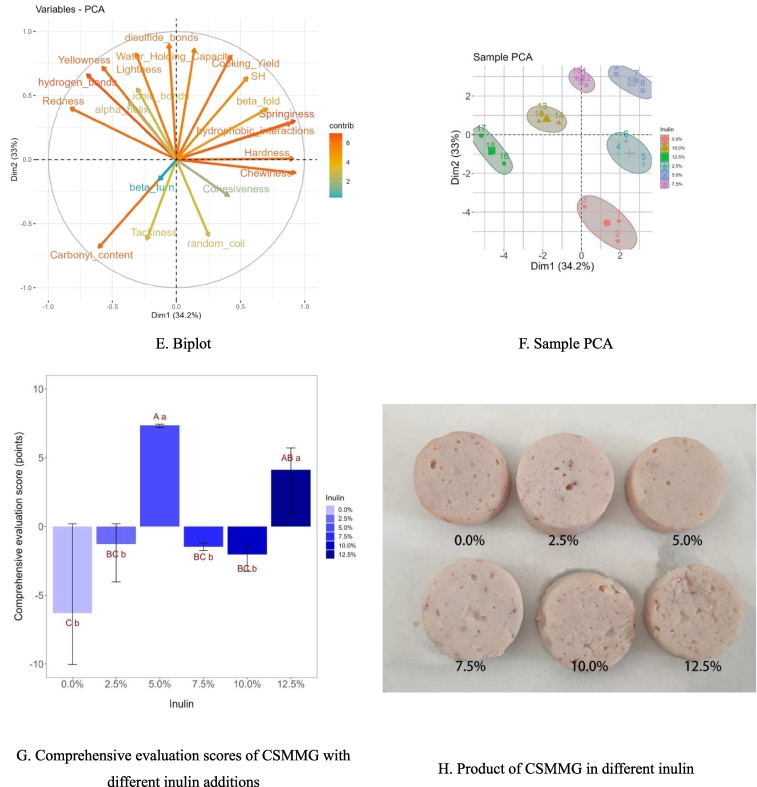

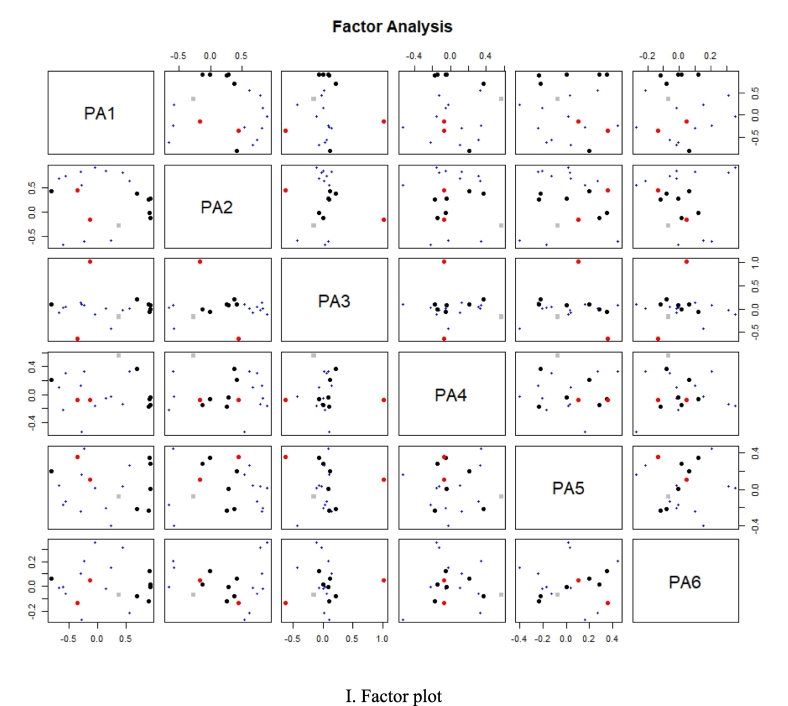
Principal component analysis plot for CSMMG.

In the study, Fig. 5 A displays the significance analysis of several indexes (inculding gel characteristics, intermolecular forces and secondary structure) of CSMMG.

In the binding ability of water [cooking yield (CY) and water holding capacity (WHC)], CY of CSMMG were positively highly significant correlation with WHC, springiness, sulfhydryl (SH) and disulfide bonds (*P* < 0.01), positively significant with lightness and beta fold (*P* < 0.05), and negatively highly significant correlation with carbonyl content (*P* < 0.01), and negatively significant with tackiness (*P* < 0.05). WHC were positively correlation with CY, lightness, disulfide bonds (*P* < 0.01) and SH (*P* < 0.05), but negatively with tackiness (*P* < 0.01).

In color (lightness, redness and yellowness), yellowness were positively correlation with lightness and redness (*P* < 0.01). Lightness, redness and yellowness were all positively correlation with hydrogen bonds (*P* < 0.01), and all negatively correlation with chewiness and random coil (*P* < 0.05).

In texture (chewiness, cohesiveness, hardness, springiness and tackiness), the three indicators (hardness, springiness and chewiness) were closely related to each other positively (*P* < 0.01) and they were positively correlation with hydrophobic interactions (*P* < 0.01) and beta fold (*P* < 0.05). In addition, springiness was closely related to SH positively (*P* < 0.01) and carbonyl content negatively (*P* < 0.01). However, among all texture indicators, tackiness was only related to CY (*P* < 0.05) and WHC (*P* < 0.01) negatively, and more specifically cohesiveness was no correlation with any other indicators (*P*>0.05).

In sulfhydryl (SH) content and carbonyl content, there was a highly significant negative correlation between the two indicators (*P* < 0.01). Interestingly, both of these were all related to CY, springiness and beta fold (*P* < 0.01), but their effects were exactly opposite.

In Molecular Forces (hydrogen bonds, hydrophobic interactions, disulfide bonds and ionic bonds), hydrogen bonds was related to ionic bonds positively (*P* < 0.05) and hydrophobic interactions negatively (*P* < 0.05). The correlation of ionic bond was not strong, only correlated with random coil negatively (*P* < 0.05). Hydrogen bonds was strongly related with color of CSMMG positively (*P* < 0.01), hydrophobic interactions was related with texture, and disulfide bonds strongly related with CY and WHC positively (*P* < 0.01).

In protein secondary structure (*β*-fold, random coil, *α*-helix, *β*-turn), *α*-helix and *β*-turn were related to each other negatively (*P* < 0.01), and uncorrelated with any other indicators (*P*>0.05). *β*-fold was positively significant correlation with springiness, SH (*P* < 0.01) and CY, hardness, chewiness (*P* < 0.05), but negatively significant with carbonyl content (*P* < 0.01). Unlike *β*-fold, random coil was negatively significant correlation with other indicators like lightness, redness, yellowness, ionic bonds and hydrogen bonds (*P* < 0.05).

The above correlation analysis shows that there is a correlation between gel characteristics (cooking yield, water holding capacity, lightness, redness, yellowness, hardness, cohesiveness, springiness, chewiness, tackiness) and main protein molecules (sulfhydryl, carbonyl content, ionic bonds, hydrogen bonds, hydrophobic interactions, disulfide bonds, beta fold, random coil, alpha helix, beta turn), and the levels of each indicator are interdependent, which provides support for further searching for principal components.

#### 3.4.2 Principal component analysis

Fig. 5 B showed the scree plot from the principal component analysis. Following principle component extraction, the total variance contributions of PC1 and PC2 were added up to 67.2 % (ESM-Table.2 and ESM-Fig. 1 A-F), and the ones of PC1, PC2, PC3, PC4 and PC5 to 90.0 % (ESM-Table.2 and ESM-Fig. 1 F), meaning that these five principal components accounted for the majority of the variation. The correlation and cluster relationship between various factors and principal components is showed in Fig. 5 C and D. The principal component analysis of the CSMMG indicators with varying inulin additions was depicted in Fig. 5 E. The length from the arrow endpoints of the rays to the origin indicated the indicator degree of contribution to the principal components, PC1 and PC2, and the analysis results were very satisfactory. The arrow endpoints of the rays represented each indicator projections in two-dimensional space. The length of the cohesiveness ray arrow from its end point to its origin was shortest. The results showed that PC1 had a positive correlation with chewiness, springiness, hardness, hydrophobic interactions (*P* < 0.01), and negative correlation with redness (*P* < 0.01). PC2 had a positive correlation with disulfide bonds,WHC, lightness, CY, yellowness, hydrogen bonds (*P* < 0.01), and negative correlation with carbonyl content and tackiness (*P* < 0.01). PC3 was positively correlated with *β*-turn, and negatively correlated with *α*-helix.

#### 3.4.3 Analysis of quality changes based on mathematical modeling and composite scores

The data results (ESM-Table. 2) indicated that the contribution rate of CSMMG quality indexes in each principal component followed a similar trend with the change of loading value. Among these, the loading value of chewiness in PC1 was the largest, with a value of 0.928 (ESM-Table. 3), indicating that the contribution of the chewiness value in PC1 was the most significant. In PC2, the absolute value of the loading value of the disulfide bonds was the largest, with a value of 0.898 (ESM-Table. 3), indicating that the contribution of the disulfide bonds in PC2 was the highest. In PC3, the loading value of *β*-turn was the largest, with a value of 0.962 (ESM-Table. 3), indicating that *β*-turn had the most significant contribution to PC3. To evaluate the quality of the gel accurately and comprehensively, the specific linear expression equations of the first five principal components were constructed using the eigenvectors of the 20 index factors as coefficients.

$${\mathrm{Dim}}_{1}=0.426\times {}_{1}+0.141\times {}_{2}–0.316\times {}_{3}–0.826\times {}_{4}–0.577\times {}_{5}+0.911\times {}_{6}+0.408\times {}_{7}+0.924\times {}_{8}+0.928\times {}_{9}–0.231\times {}_{10}+0.554\times {}_{11}–0.605\times {}_{12}–0.307\times {}_{13}–0.696\times {}_{14}+0.890\times {}_{15}–0.058\times {}_{16+}0.708\times {}_{17}+0.253\times {}_{18}–0.368\times {}_{19}–0.135\times {}_{20}$$

$${\mathrm{Dim}}_{2}=0.810\times {}_{1}+0.864\times {}_{2}+0.828\times {}_{3}+0.402\times {}_{4}+0.723\times {}_{5}+0.007\times {}_{6}–0.283\times {}_{7}+0.302\times {}_{8}–0.102\times {}_{9}–0.623\times {}_{10}+0.641\times {}_{11}–0.685\times {}_{12}+0.555\times {}_{13}+0.664\times {}_{14}+0.284\times {}_{15}+0.898\times {}_{16}+0.397\times {}_{17}–0.595\times {}_{18}+0.449\times {}_{19}–0.157\times {}_{20}$$

$${\mathrm{Dim}}_{3}=-0.033\times {}_{1}–0.009\times {}_{2}+0.134\times {}_{3}+0.100\times {}_{4}+0.029\times {}_{5}–0.055\times {}_{6}–0.236\times {}_{7}+0.091\times {}_{8}+0.013\times {}_{9}+0.094\times {}_{10}+0.002\times {}_{11}+0.051\times {}_{12}+0.131\times {}_{13}–0.085\times {}_{14}+0.100\times {}_{15}–0.115\times {}_{16}+0.212\times {}_{17}–0.483\times {}_{18}–0.668\times {}_{19}+0.962\times {}_{20}$$

$${\mathrm{Dim}}_{4}=-0.117\times {}_{1}–0.038\times {}_{2}+0.159\times {}_{3}+0.232\times {}_{4}+0.313\times {}_{5}–0.085\times {}_{6}+0.698\times {}_{7}–0.046\times {}_{8}–0.155\times {}_{9}+0.351\times {}_{10}+0.306\times {}_{11}–0.208\times {}_{12}–0.570\times {}_{13}+0.120\times {}_{14}–0.159\times {}_{15}–0.127\times {}_{16}+0.376\times {}_{17}–0.070\times {}_{18}–0.099\times {}_{19}–0.008\times {}_{20}$$

$${\mathrm{Dim}}_{5}=0.036\times {}_{1}–0.234\times {}_{2}–0.258\times {}_{3}+0.189\times {}_{4}–0.126\times {}_{5}+0.344\times {}_{6}–0.076\times {}_{7}+0.009\times {}_{8}+0.281\times {}_{9}+0.511\times {}_{10}+0.281\times {}_{11}–0.193\times {}_{12}+0.171\times {}_{13}+0.035\times {}_{14}–0.217\times {}_{15}+0.010\times {}_{16}–0.214\times {}_{17}–0.453\times {}_{18}+0.382\times {}_{19}+0.064\times {}_{20}$$

Using the above formula, calculate the factor score(ESM-Table. 4). Based to the variance and cumulative variance contributions of principal components (ESM-Table. 2–4), a mathematical operation model of the comprehensive evaluation score *Y* (Fig. 5 F,G,I) of chicken and squilla mince mixtures gel (CSMMG) was established.

$$Y=0.380{\mathrm{Dim}}_{1}+0.367{\mathrm{Dim}}_{2}+0.100{\mathrm{Dim}}_{3}+0.084{\mathrm{Dim}}_{4}+0.069{\mathrm{Dim}}_{5}$$

Finally, the data of each group were averaged to obtain the comprehensive score, as shown in Fig. 5 G. According to PCA (Fig. 5 F—I), when the inulin content was 5.0 %, the quality of CSMMG was the best.

### 3.5 Molecular docking

**Fig. 6:**
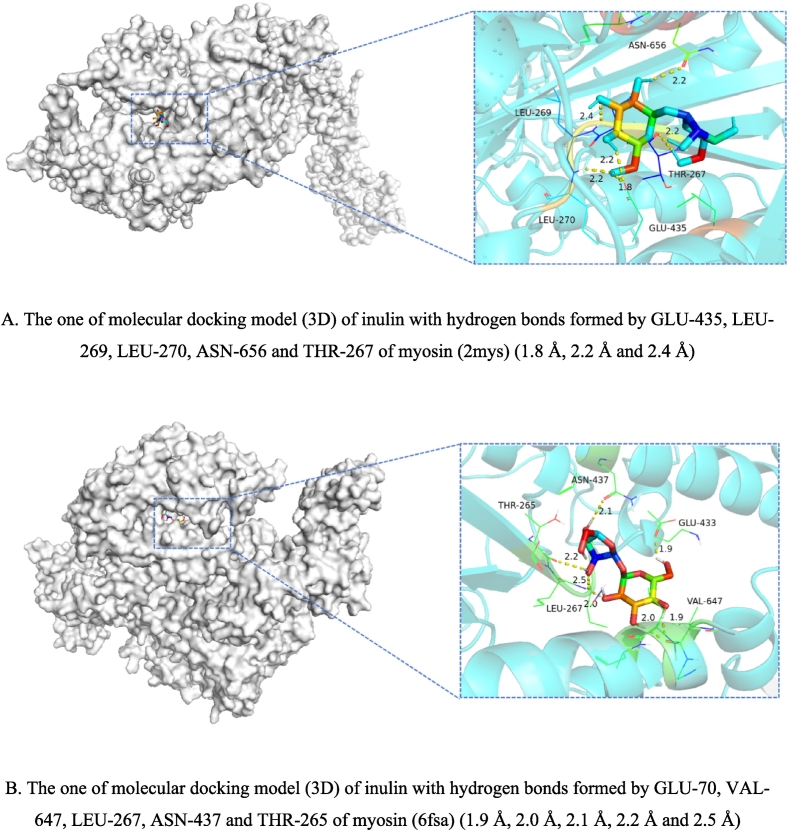
Molecular docking of myosin and inulin.

Myosin heavy chain (MHC) and paramyosin (PM) have been proven to be the major protein in chicken squilla mince mixture gel (CSMMG) in our research 3.2.2. Based on previous speculation, myosin might be likely to be selected as the receptor to bind with inulin. So the molecular docking of myosin with inulin were simulated for prediction by software *AutoDock 1.5.7*. In the docking state, inulin could form some stable complexs with myosin. Among these complexs, two major complexs of the lower binding energy than the others were shown by the molecular docking 3D results (Fig. 6), with a selected binding free energy of - 3.61 KJ/mol and − 2.30 KJ/mol. As shown in Fig. 6 A, hydrophobic interaction (hydrogen bonds) exist between residues GLU-435, LEU-269, LEU-270, ASN-656 and THR-267 of myosin (2mys) and inulin, while the bond lengths is 1.8 Å, 2.2 Å and 2.4 Å. As shown in Fig. 6 B, hydrogen bonds exist between residues GLU-70, VAL-647, LEU-267, ASN-437 and THR-265 of myosin (6fsa) and inulin, while the bond lengths is 1.9 Å, 2.0 Å, 2.1 Å, 2.2 Å and 2.5 Å. Studies have shown that inulin can form hydrogen bonds with trp, Arg, Ile, Gly, Ser, Phe, and Asn amino acids on nitric oxide synthase (Hashmi et al., 2024). And this study shows that inulin can also form hydrogen bonds with Glu, Leu, Thr and Val amino acids. It is well showing how small molecules (inulin) and proteins (myosin) bind together, as well as their strength of interaction. Above all of these, it has been demonstrated that the stability of the complexes depends on hydrogen bonding and hydrophobic interactions between the myosin and inulin, consistent with the results of PCA in our study.

**Fig. 7:**
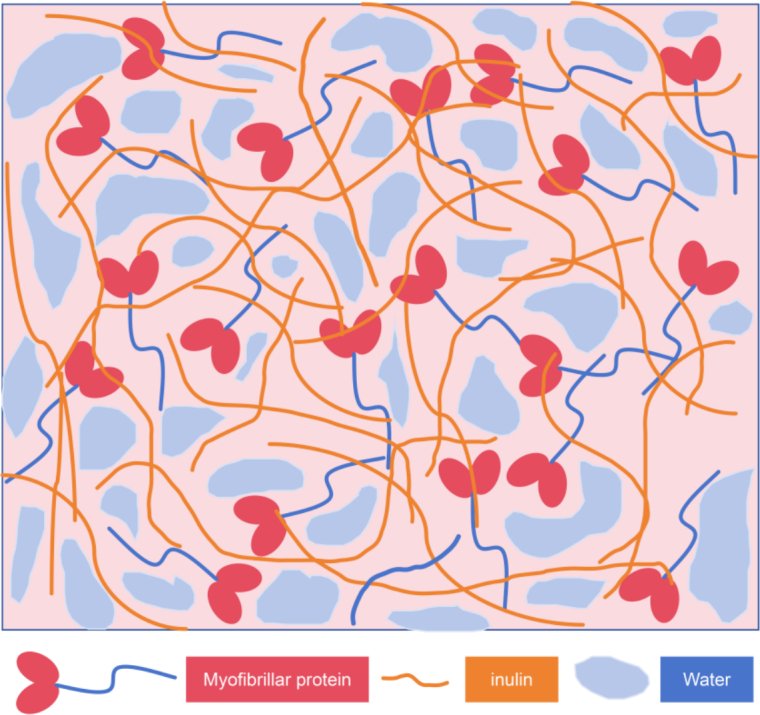
Schematic diagram of the interaction mechanism between inulin and myofibrillar protein.

Through the above experiments, inulin strengthened the structure by increasing the sulfhydryl content, slowing down the carbonyl content, and promoting the formation of hydrogen bond, disulfide bond, *β*-fold and hydrophobic interaction of CSMMG for making the gel structure more stable (Fig. 7), thus to obtain higher sensory scores. Maybe inulin has a strong antioxidant protective effects on myoglobin and other substances (Ma et al., 2022). Under heating conditions, inulin undergoed a certain degree of Maillard reaction with proteins (Hemmati et al., 2022; Serdaroğlu et al., 2016). Inulin is a multi-purpose water-soluble dietary fiber, so excess of it could be swelled by absorbing water. The excessive expansion of inulin could expand the protein gap, block myofibrillar protein formation, destroy the stability of gel, and cause the hardness, springiness and chewiness of CSMMG to decrease and the stickiness to increase (Wang et al., 2024). Inulin promoted the formation of stable gel structure, through reducing protein aggregation and making the microstructure compact and uniform (Fig. 7) (Li et al., 2024; Tao et al., 2023; Wu et al., 2023). Inulin also obviously enhanced the gelling behavior of myofibrillar protein (MP) molecules with a denser and more uniform network structure by the hydrogen bonding between the myosin (residues GLU-435, LEU-269, LEU-270, ASN-656, THR-267 or residues GLU-70, VAL-647, LEU-267, ASN-437, THR-265) and inulin and hydrophobic interactions, to exhibit the highest cooking yield and the best textural characteristics for processing meat protein gel products.

## 4 Conclusions

The findings demonstrated that adding 5.0 % inulin could significantly improve the cooking yield, water-holding property, color value, hardness, elasticity, chewiness, sensory score, microstructural denseness, MHC, and PM retention of CSMMG. It could also result in the CSMMG having the best protein structural stability, with a more compact spatial structure, a brighter color, a more resilient texture, and a higher acceptability. On the other hand, adding too much inulin would be harmful to the gel formation process and lower the sensory quality of the gels. Additionally, Inulin had the certain delaying effect on the protein oxidation to promote the formation and hydrophobic interaction of sulfhydryl group, hydrogen bond, disulfide bond, *α*-helix, and reduce the carbonyl content, but had no significant effect on the ion bond of CSMMG. The length of the cohesiveness ray arrow from its end point to its origin was shortest. According to PCA, the results showed that PC1 was positive correlation with chewiness, springiness, hardness, hydrophobic interactions, and negative correlation with redness. PC2 was positive correlation with disulfide bonds,WHC, lightness, CY, yellowness, hydrogen bonds, and negative correlation with carbonyl content and tackiness. PC3 was negatively correlated with cooking yield, hardness, and chewiness, and positively correlated with other indicators. Inulin also obviously enhanced the gelling behavior of myofibrillar protein (MP) molecules with a denser and more uniform network structure by the hydrogen bonding between the myosin (residues GLU-435, LEU-269, LEU-270, ASN-656, THR-267 or residues GLU-70, VAL-647, LEU-267, ASN-437, THR-265) and inulin, to exhibit the highest cooking yield and the best textural characteristics for processing meat protein gel products. The quality of CSMMG was the best with 5.0 % inulin. In summary, the inclusion of inulin could facilitate the development of a protein gel network in the CSMMG and improve the processing performance and sensory quality of gel. This study will offer a theoretical basis for future research and development of dietary fiber-enriched low-fat sausage and the improvement of the quality of mixture meat products.

## Funding

This work was supported by “Liaoning Province Applied Basic Research Program (20230242)”, “Liaoning Provincial College Student Innovation and Entrepreneurship Project (S202310160003X)” and “College Student Innovation and Entrepreneurship Project of Jinzhou Medical University (X202410160009X)”.

## Institutional review board statement

The study was conducted in accordance with the Declaration of Helsinki, and approved by the Ethics Committee of Jinzhou Medical University (Approved on 29 September 2022).

## CRediT authorship contribution statement

**Lijing Geng:** Writing – original draft, Validation, Supervision, Methodology, Investigation, Conceptualization. **Jing Liang:** Validation, Methodology, Conceptualization. **Dan Wang:** Resources, Data curation. **Wei Huang:** Resources, Data curation. **Hang Fu:** Resources, Data curation. **Mbinga Isequias Elamba Tertulliano:** Resources, Data curation. **Muhammad Zain Ul Aabideen:** Formal analysis. **Wei Zhou:** Supervision, Formal analysis. **Quimbamba Silvia Jacinta Calombe:** Formal analysis. **Aqsa Shafique:** Writing – review & editing.

## Declaration of competing interest

The authors declare that they have no known competing financial interests or personal relationships that could have appeared to influence the work reported in this paper.

## Data Availability

Data will be made available on request.

## Appendix A Supplementary data

Supplementary material 1 Image 1

Supplementary material 2 Image 2
