# Supplementary material for: Gel properties of chicken-squilla low-fat mince gel with inulin: Physicochemical properties, microstructure, intermolecular interactions, and formation mechanism
Source: Food Chem X. 2025 Mar 17;27:102347. doi: 10.1016/j.fochx.2025.102347 (PMC11985139; doi:10.1016/j.fochx.2025.102347)
Supplement: Supplementary file 1 — Supplementary material 1 [file mmc1.docx]

Electronic Supplementary Material (ESM)

| **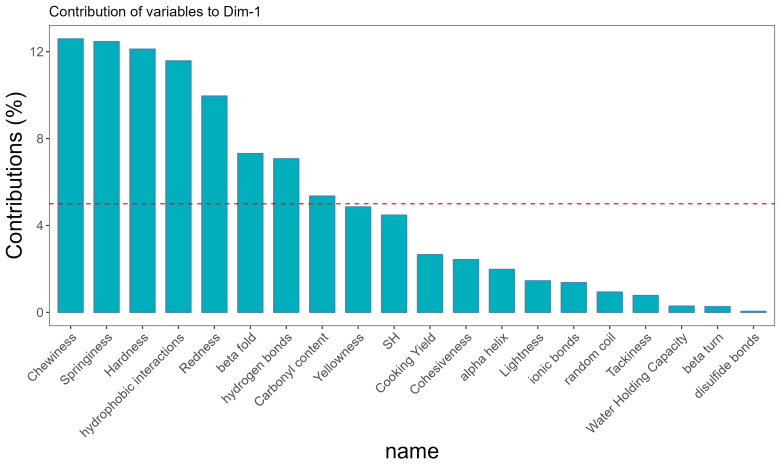** | 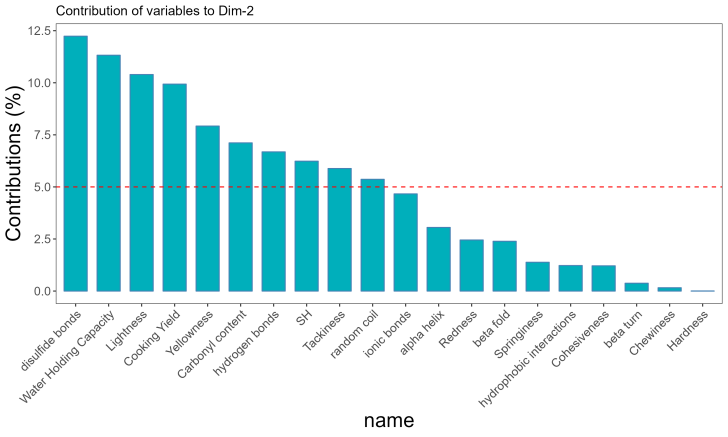 |
| --- | --- |
| A.Contribution of variables to PC1 | B.Contribution of variables to PC2 |
| 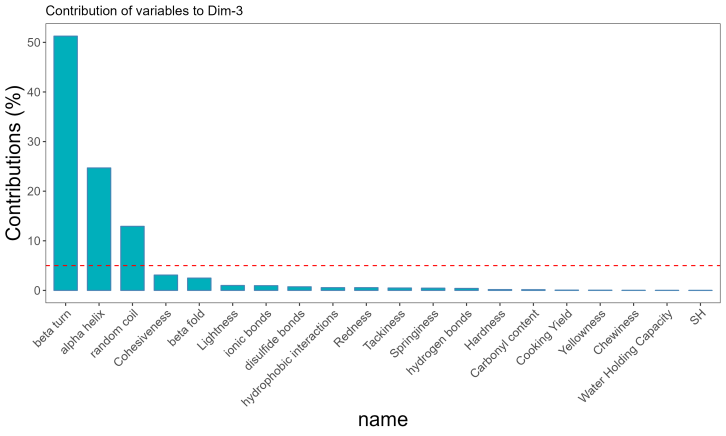 | 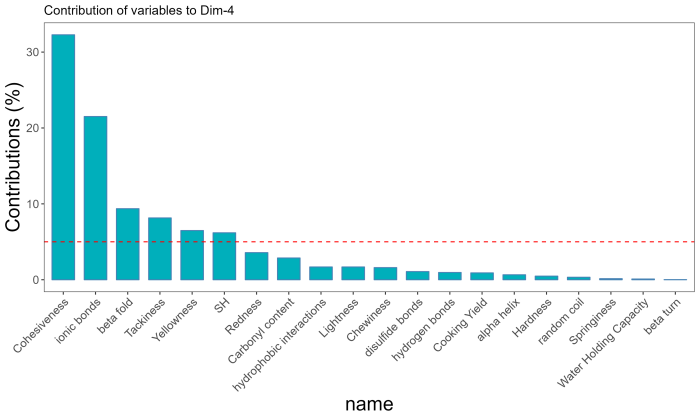 |
| C.Contribution of variables to PC3 | D.Contribution of variables to PC4 |
| 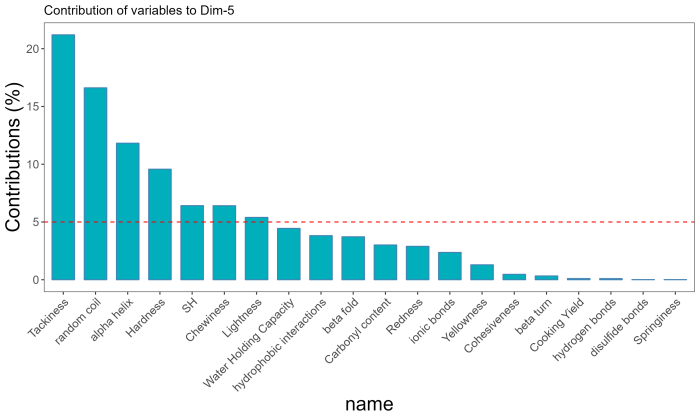 | 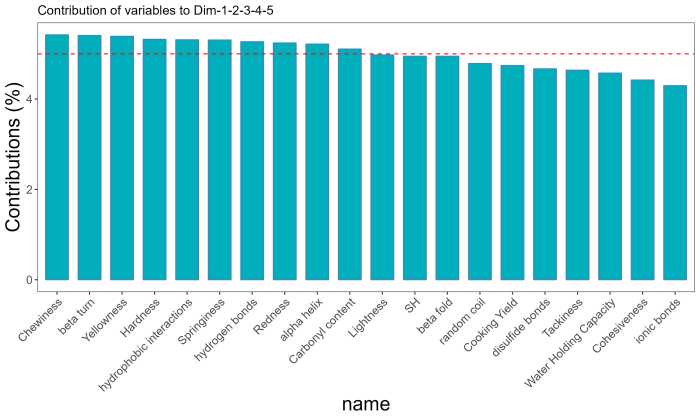 |
| E.Contribution of variables to PC5 | F.Contribution of variables to PC1, PC2, PC3, PC4 and PC5 |
| ESM-Fig.1 Contribution of variables | |

ESM-Table. 1 Sensory evaluation system (scores) of CSMMG

| **Classification** | **Sensory evaluation standard** | **scores** | |
| --- | --- | --- | --- |
| Color | The color distribution of the product is very uniform with good luster | 8~10 | |
|  | The color distribution of the product is relatively uniform and shiny. | 4~7 | |
|  | The color distribution of the product is uneven and the luster is poor. | 1~3 | |
| Appearance | The cross-section of the product is very compact and flat with no pores and no juice exudation. | 8~10 | |
|  | The cross-section of the product is relatively flat with a small amount of pores and a small amount of juice exudation. | 4~7 | |
|  | The cross-section of the product is loose and rough with many and uneven pores and a large amount of juice exudation. | 1~3 | |
| Flavour | With rich raw material flavor and no peculiar smell. | 8~10 | |
|  | With very light raw material flavor or with slightly odorous. | 4~7 | |
|  | With no raw material fragrance or with odorous. | 1~3 | |
| Mouth feel | Good elasticity, good chewiness | 8~10 | |
|  | General elasticity, general chewiness | 4~7 | |
|  | Poor elasticity, too hard or too soft texture | 1~3 | |
| Overall acceptability | Good acceptability | 8~10 | |
|  | Acceptability is general. | 4~7 | |
|  | Poor acceptability | | 1~3 |

ESM-Table. 2 Initial eigenvalues, contribution rates of variance and cumulative contribution rates of variance of principal components

|  | **Initial eigenvalues** | **Contribution rates of variance (%)** | **Cumulative contribution rates of variance (%)** |
| --- | --- | --- | --- |
| PC1 | 6.846 | 34.228% | 34.228% |
| PC2 | 6.598 | 32.990% | 67.218% |
| PC3 | 1.806 | 9.032% | 76.250% |
| PC4 | 1.510 | 7.552% | 83.802% |
| PC5 | 1.233 | 6.164% | 89.966% |
| PC6 | 0.606 | 3.032% | 92.998% |
| PC7 | 0.394 | 1.969% | 94.968% |
| PC8 | 0.291 | 1.454% | 96.421% |
| PC9 | 0.215 | 1.076% | 97.497% |
| PC10 | 0.166 | 0.831% | 98.328% |

ESM-Table. 3 Eigenvectors for principal components

| **Variable** | **Index** | **PC1** | **PC2** | **PC3** | **PC4** | **PC5** |
| --- | --- | --- | --- | --- | --- | --- |
| X_1_ | Cooking_Yield | 0.426 | 0.810 | -0.033 | -0.117 | 0.036 |
| X_2_ | Water_Holding_Capacity | 0.141 | 0.864 | -0.009 | -0.038 | -0.234 |
| X_3_ | Lightness | -0.316 | 0.828 | 0.134 | 0.159 | -0.258 |
| X_4_ | Redness | -0.826 | 0.402 | 0.100 | 0.232 | 0.189 |
| X_5_ | Yellowness | -0.577 | 0.723 | 0.029 | 0.313 | -0.126 |
| X_6_ | Hardness | 0.911 | 0.007 | -0.055 | -0.085 | 0.344 |
| X_7_ | Cohesiveness | 0.408 | -0.283 | -0.236 | 0.698 | -0.076 |
| X_8_ | Springiness | 0.924 | 0.302 | 0.091 | -0.046 | 0.009 |
| X_9_ | Chewiness | 0.928 | -0.102 | 0.013 | -0.155 | 0.281 |
| X_10_ | Tackiness | -0.231 | -0.623 | 0.094 | 0.351 | 0.511 |
| X_11_ | SH | 0.554 | 0.641 | 0.002 | 0.306 | 0.281 |
| X_12_ | Carbonyl_content | -0.605 | -0.685 | 0.051 | -0.208 | -0.193 |
| X_13_ | ionic_bonds | -0.307 | 0.555 | 0.131 | -0.570 | 0.171 |
| X_14_ | hydrogen_bonds | -0.696 | 0.664 | -0.085 | 0.120 | 0.035 |
| X_15_ | hydrophobic_interactions | 0.890 | 0.284 | 0.100 | -0.159 | -0.217 |
| X_16_ | disulfide_bonds | -0.058 | 0.898 | -0.115 | -0.127 | 0.010 |
| X_17_ | beta_fold | 0.708 | 0.397 | 0.212 | 0.376 | -0.214 |
| X_18_ | random_coil | 0.253 | -0.595 | -0.483 | -0.070 | -0.453 |
| X_19_ | alpha_helix | -0.368 | 0.449 | -0.668 | -0.099 | 0.382 |
| X_20_ | beta_turn | -0.135 | -0.157 | 0.962 | -0.008 | 0.064 |

ESM-Table. 4 Factor score

|  | **PC1** | **PC2** | **PC3** | **PC4** | **PC5** |
| --- | --- | --- | --- | --- | --- |
| Cooking_Yield | 4.931 | -11.125 | -1.611 | -1.600 | -1.154 |
| Water_Holding_Capacity | 4.759 | -13.717 | -1.656 | -0.296 | -0.809 |
| Lightness | 0.419 | -9.277 | 3.068 | -2.526 | 0.576 |
| Redness | 4.861 | -2.479 | 2.206 | -0.167 | 1.602 |
| Yellowness | 8.162 | -3.770 | -1.374 | 4.166 | -0.025 |
| Hardness | 5.862 | -0.836 | -0.326 | 0.514 | 0.629 |
| Cohesiveness | 7.238 | 6.960 | -2.481 | -0.915 | 0.659 |
| Springiness | 8.053 | 5.697 | 2.194 | 1.243 | 0.648 |
| Chewiness | 4.548 | 7.822 | 0.007 | -1.363 | 2.291 |
| Tackiness | -0.618 | 7.312 | -1.585 | -1.274 | -0.920 |
| SH | 0.095 | 7.246 | -1.039 | -1.299 | -1.139 |
| Carbonyl_content | 0.660 | 6.378 | 0.194 | 0.179 | -2.158 |
| ionic_bonds | -5.150 | 2.738 | 1.235 | 0.219 | -1.507 |
| hydrogen_bonds | -2.955 | 1.521 | 2.703 | 1.554 | -1.231 |
| hydrophobic_interactions | -5.563 | 1.743 | 1.226 | -0.062 | -0.290 |
| disulfide_bonds | -10.173 | -3.703 | 1.142 | 0.460 | 0.246 |
| beta_fold | -13.152 | -0.119 | -1.894 | -0.180 | 0.850 |
| random_coil | -11.978 | -2.389 | -2.010 | 1.348 | 1.732 |
| alpha_helix | 4.931 | -11.125 | -1.611 | -1.600 | -1.154 |
| beta_turn | 4.759 | -13.717 | -1.656 | -0.296 | -0.809 |
